# Supplementary material for: Pyruvate Dehydrogenase A1 Phosphorylated by Insulin Associates with Pyruvate Kinase M2 and Induces LINC00273 through Histone Acetylation
Source: Biomedicines. 2022 May 27;10(6):1256. doi: 10.3390/biomedicines10061256 (PMC9220252; doi:10.3390/biomedicines10061256)
Supplement: Supplementary file 1 [file biomedicines-10-01256-s001.zip › biomedicines-1715893-supplementary.pdf]

A

Mouse Pyruvate kinase M2 (PKM2) (NM_011099.3)

1	MPKPDSEAGT	AFIQTQQLHA	AMADTFLEHM	CRLDIDSAPI	TARNTGIICT
51	IGPASRSVEM	LKEMIKSGMN	VARLNFSHGT	HEYHAETIKN	VRAATESFAS
101	DPILYRPVAV	ALDTKGPEIR	TGLIKGSGTA	EVELKKGATL	KITLDNAYME
151	KCDENILWLD	YKNICKVVEV	GSKIYVDDGL	ISLQVKEKGA	DYLVTEVENG
201	GSLGSKKGVN	LPGAAVDLPA	VSEKDIQDLK	FGVEQDVDMV	FASFIRKAAD
251	VHEVRKVLGE	KGKNIKIISK	IENHEGVRRF	DEILEASDGI	MVARGDLGIE
301	IPAEKVFLAQ	KMMIGRCNRA	GKPVICATQM	LESMIKKPRP	TRAEGSDVAN
351	AVLDGADCIM	LSGETAKGDY	PLEAVRMQHL	IAREAEAAIY	HLQLFEELRR
401	LAPITSDPTE	AAAVGAVEAS	FKCCSGAIIV	LTKSGRSAHQ	VARYRPRAPI
451	IAVTRNPQTA	RQAHLYRGIF	PVLCKDAVLD	AWAEDVDLRV	NLAVNVGKTR
501	GFFKKGDVVI	VLTGWRPGSG	FTNTMRVVPV	P	

B

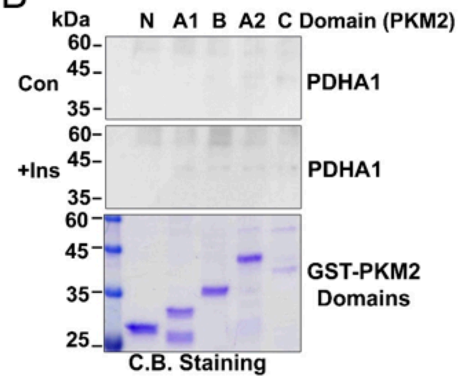

Figure S1. Identified peptides of PKM2 by MALDI-TOF analysis. (A) Amino acid sequence of mouse PKM2 is shown and the peptide fragments of PKM2 (NM_011099.3) that were identified with MADLI-TOF (red color). The peptide fragment underlined indicates the difference from that of PKM1. Peptides in red indicate the ones identified by MALDI-TOF analysis (eBiogene). (B) Insulin (100 nm for 48 h) or without insulin HepG2 cells lysate were incubated with each recombinant GST-PKM2 domains (2 μg protein) and PDHA1 level by western blotting (upper panel). GST-PKM2 domains were visualized by Coomassie-blue staining (lower panel).

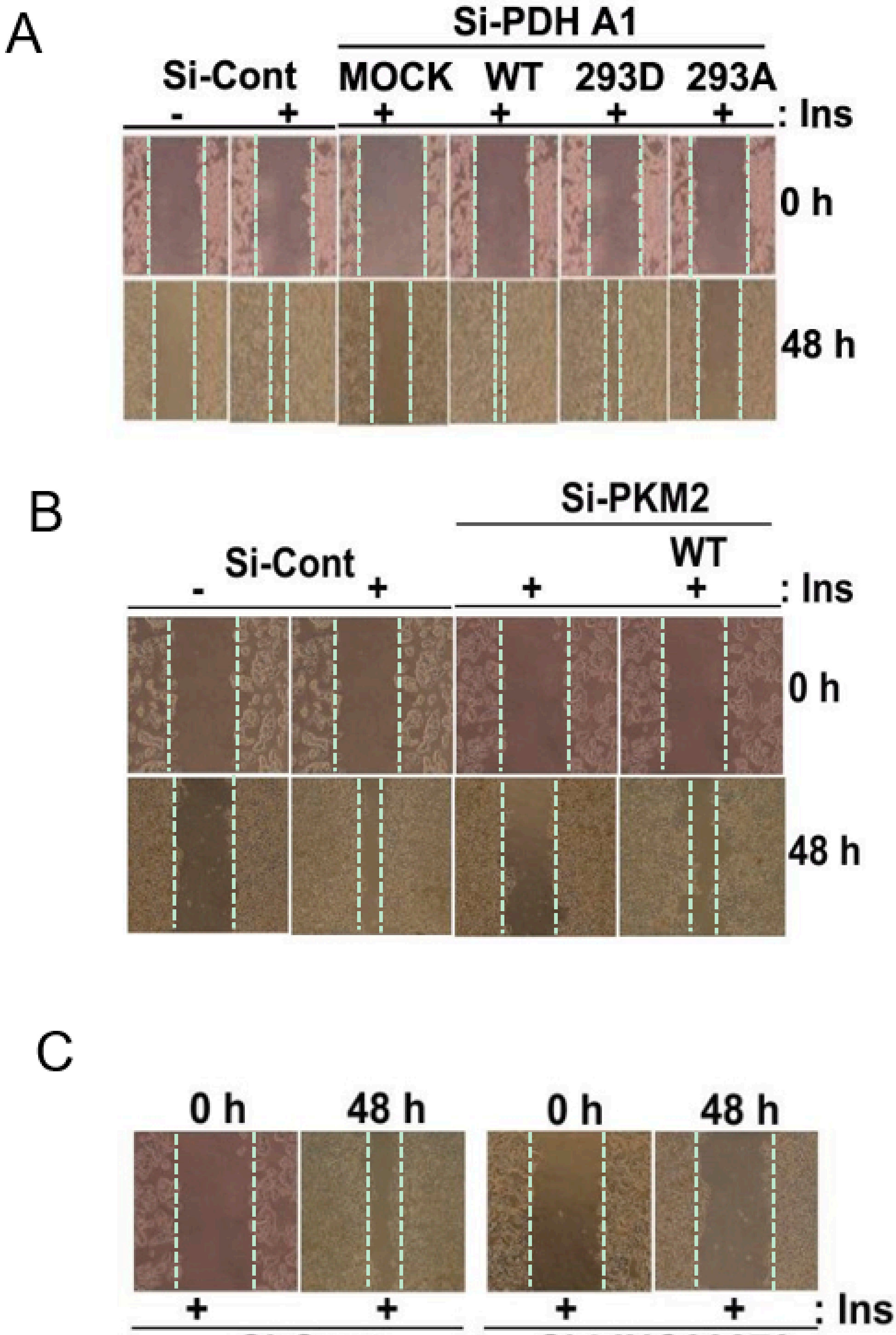

Figure S2. Cell migration by wound healing assay. (A) si-PDHA1 transfection and addback with PDHA1 WT, S293D and S293A to the HepG2 cells which were stimulated with insulin (100 nM for 48 h) and cell migration was measured by the wound healing assay (B) HepG2 cells were transfected with si-PKM2 and in presence of PKM2 WT addback, cell migration upon insulin (100 nM for 48 h) was measured by wound healing assay. (C) HepG2 cells were transfected with si-LINC00273 and cell migration was determined by the wound healing assay followed by insulin (100 nM for 48 h).

A Liver Cancer

Number	Band Intensity of p-PDH	Band Intensity of PKm2	Age	Date Of Diagnosis	Date Of Treatment	Date Of Death	Treatment Consistency	Gender
1	9.98	44.1	75	2012-12-11	2014-01-27	2014-11-25	Dead	Male
2	10.45	4.9	72	2011-12-30	2017-03-23	2017-05-19	Dead	Male
3	2.08	17.15	59	2013-08-05	2014-07-16		Cured	Male
4	3.71	26.13	61	2011-04-29	2018-09-09	2018-09-09	Dead	Male
5	4.17	26.95	55	2013-07-18	2016-07-05	2016-07-05	Dead	Female
6	3.24	23.68	47	2014-07-22	2016-03-12	2016-03-12	Dead	Female
7	19.03	9.8	68	2016-11-10-	2020-08-19		Continue	Male
8	19.49	22.05	63	2017-02-09	2017-10-02	2017-10-02	Dead	Male
9	20.66	8.16	61	2017-02-10	2020-07-21		Continue	Male
10	23.67	7.35	49	2017-03-14	2020-08-06		Continue	Male
11	9.74	15.51	62	2017-04-25	2017-09-15	2018-05-28	Dead	Male
12	16.71	6.53	66	2017-05-15	2020-06-01		Continue	Male
13	1.62	27.76	64	2015-09-07	2020-07-06		Continue	Male
13	1.72	22.5	64	2015-09-07	2020-07-06		Continue	Male
14	8.74	10.5	72	2016-07-07	2020-05-12		Continue	Male
15	12.62	8	62	2018-09-10	2020-08-11		Continue	Male
16	6.99	12.5	55	2018-10-15	2020-06-08		Continue	Male
17	2.62	6	56	2018-11-22	2020-08-21		Continue	Male
18	5.99	6.5	71	2018-12-28	2020-08-10		Continue	Male
19	6.37	24.5	64	2019-01-04	2020-08-04		Continue	Female
20	9.49	7.5	56	2014-09-30	2019-04-18		Cured	Male
21	11.5	5	76	2019-03-08	2020-07-13		Continue	Female
22	9.49	16.5	74	2019-03-07	2019-09-19		Cured	Male
23	5.62	13.5	74	2019-03-11	2020-08-05		Continue	Male
24	2.75	4	56	2019-03-05	2020-08-07		Continue	Male
25	1	1	46	2019-07-16	2020-03-23		Continue	Male

B Lung Cancer

Number	Band Intensity of p-PDH	Age	Cancer Stage	Date Of Diagnosis	Date Of Treatment	Date Of Death	Treatment Consistency	Gender
1	12.5	74	Unknown	2012-05-10	2015-04-28	2016-01-07	Dead	Male
2	1.4	21	I	2014-03-06	2018-05-03		Cured	Female
3	12.7	73	II	2014-06-16	2015-04-16	2015-04-25	Dead	Male
4	0	75	II	2014-07-21	2020-08-19		Continue	Female
5	4.2	33	III	2015-08-06	2020-08-03		Continue	Male
6	0	69	III	2015-09-30	2020-06-26		Continue	Male
7	12.3	56	III	2016-02-26	2016-06-29	2016-06-29	Dead	Male
8	1.8	57	II	2016-03-24	2020-08-25		Continue	Male
9	1.8	46	I	2016-03-10	2020-08-20		Continue	Female
10	3.8	77	I	2016-04-27	2019-08-12		Cured	Female
11	1	60	III	2016-05-20	2017-04-17	2017-04-17	Dead	Male
12	3.2	62	II	2016-11-13	2020-04-07		Continue	Male
13	5.1	70	I	2016-11-15	2017-02-20	2017-02-20	Dead	Male
13	4	70	I	2016-11-15	2017-02-20	2017-02-20	Dead	Male
14	2.1	60	I	2016-11-09	2019-10-29		Cured	Male
15	11.3	63	II	2016-11-15	2018-05-10	2018-05-10	Dead	Female
16	0	66	III	2016-11-18	2020-06-29		Continue	Female
17	0	56	II	2016-12-15	2020-04-24		Continue	Female
18	2.4	58	I	2016-09-29	2020-06-29		Continue	Female
19	0	57	I	2017-01-11	2020-06-18		Continue	Female
20	3.1	78	I	2016-08-11	2020-05-26		Continue	Male
21	1	64	I	2016-12-30	2020-07-09		Continue	Female
22	2.9	78	III	2016-11-26	2019-07-17		Continue	Female
23	3.9	55	I	2017-01-16	2018-05-11		Cured	Female
24	2.7	76	II	2017-02-15	2020-08-12		Continue	Male
25	2.5	67	I	2017-01-31	2020-08-17		Continue	Male

Figure S3. liver and lung cancer patient's information. (A,B). Total Information on death and survival of the patients were obtained from the Korean Statistical Information Service KOSTAT database (KOSIS) of the Korean government and approved by the institutional review board (IRB: HIRB-2019-048) of Hallym University (Chuncheon, Korea).
